# 

**Published:** 1883-04

**Authors:** 


### NEW BOOKS.

HANDBOOK OF INVERTEBRATE ZOOLOGY. For Laboratories and Seaside work. By W. K. Brooks, Ph. D. S. E. Cassino, 32 Hawley street, Boston, Mass., publisher. Price $3,00.

This work is designed more particularly for the amateur investigator of animal life. It deals with the various types of the lower orders of animal structure, giving their macroscopic and microscopic anatomy, and illustrating the same by means of numerous well executed cuts from original drawings of the author. Methods are also given for collecting, preserving and examining infusorial life. Excellent drawings are given of the anatomical structure of the Vorticellæ, Sponge, Campanularian Hydroid, Hydro-Medusa, Star Fish, Sea Urchin, Earth worm, Leech, Crab, Cyclops,

Grasshopper, Unio and Squid. The book is a large 8 vo. of 400 pages, excellently printed, on good paper. We regard it as an excellent aid to the student in biology.

---

ANATOMICAL TECHNOLOGY, as applied to the Domestic Cat: An introduction to human, veterinary and comparative Anatomy. By Burt G. Wilder, B. S., M. D., and Simon H. Gage, B. S. The first, Professor of Physiology comparative Anatomy and Zoology in Cornell University; the latter assistant Professor of Physiology and lecturer on Microscopical Technology in the same institution. A. S. Barnes & Company, New York and Chicago, publishers. Price ———.

This is an admirable treatise upon the theme amply divulged in the title of the work, as above given. A work of this character, written by Professors Wilder and Gage, is a sufficient guaranty of its value. Both these gentlemen have been dilligent and very capable explorers in anatomical technology, and have from time to time published much that is new in the science. Most elaborate papers upon the anatomy of the cat have heretofore been published by various scientific societies, from the pen of Dr. Wilder, while equally valuable articles upon the anatomy and physiology of the menobranchus have appeared as the result of Prof. Gage's investigations. This splendid work could not well be bettered in the method of instruction adopted. It is a great pity that the publishers have executed their work so miserably. The paper is thin and poor, while the illustrations are simply abominable.

---

PHYSIOLOGY AND PATHOLOGY OF THE BLOOD.—Comprising the origin, mode of development, pathological and post-mortem changes of its morphological elements in mamalian and oviparous vertebrates. By Richard Norris, M.D., F. R. S. E., Prof. of physiology in Queen's College, Birmingham, Eng. Smith, Elder & Co., 15 Waterloo Place, London Eng. Price, $7.00.

This is a treatise elaborately illustrated with micro-photographic plates, to prove the existence of a third blood corpuscle which has the same color and is of the same refractive index as the liquor sanguinis, therefore invisible. It is claimed that this new corpuscle, which he names the phantom corpuscle, differs in every essential particular from the red corpuscle, or the corpuscles of the lymph and spleen. He claims that this new element gives rise to fibrin, and is the direct cause of the coagulation of the blood.

He describes different methods of examining these corpuscles: By packing, in which the corpuscles are compressed under a cover glass by means of which they are made to lie flatwise and in single layers when the red corpuscles mould themselves around the phantom ones, revealing their presence. By altering the refractive index of the liquor sanguinis; this is done by placing a drop of saturated solution of salt upon the end of the finger and pricking through it, permitting the blood to mingle with the salt, when, adopting the "packing" method, the outlines of the phantoms are seen because of the modified refractive power of the liquor sanguinis: By isolation; this is a device for drawing off the liquor sanguinis into a different compartment in the cover glass, permitting the different corpuscles to adhere to the glass, when their form and size are readily made out from the interstices surrounding the visible corpuscles. The apparatus used is clumsy and uncertain in its action. It is manifest that the author has never heard of the Holman current slide—made for this very purpose, else would he have used it and spared himself much trouble.

Several other methods are recommended for showing these phantom corpuscles; as by spreading and drying; by tinting the liquor sanguinis; by staining the invisible corpuscle, etc.

Micro-photographs are given, in great profusion, of the "invisible corpuscle," under their various forms of treatment.

How much of the results obtained are due to the methods employed must be determined by other experimenters in the same field. The book is rather a costly one, but is worth its price to those interested in the study of the blood. It is well printed, on good paper, and with excellent illustrations.

---

AMERICAN SOCIETY OF MICROSCOPISTS.—The next meeting of this society will transpire at Chicago, early in August. Secretary Kellicott already reports a large number of papers that will be read upon that occasion. This society embraces most, if not all, of the prominent microscopists of the country, so that the meetings are of great interest and value to those studying the science.

